# Relationship between Expression of Chalcone Synthase Genes and Chromones in Artificial Agarwood induced by Formic Acid Stimulation Combined with *Fusarium* sp. A2 Inoculation

**DOI:** 10.3390/molecules22050686

**Published:** 2017-04-25

**Authors:** Xiaodong Chen, Xiaoling Zhu, Meirou Feng, Zhaojian Zhong, Xin Zhou, Xiaoying Chen, Wei Ye, Weimin Zhang, Xiaoxia Gao

**Affiliations:** 1 State Key Laboratory of Applied Microbiology Southern China, Guangdong Provincial Key Laboratory of Microbial Culture Collection and Application, Guangdong Open Laboratory of Applied Microbiology, Guangdong Institute of Microbiology, Guangzhou 510070, China; vchenxiaodong0823@163.com (X.C.); yewei@gdim.cn (W.Y.); 2 School of Pharmacy, Guangdong Pharmaceutical University, Guangzhou 510006, China; workzhu@foxmail.com (X.Z.); meirou0330@163.com (M.F.); zhongzhaojian88@163.com (Z.Z.); zhouxin0316@163.com (X.Z.); gycxy00@163.com (X.C.); 3 Sirio Pharma Co., Ltd., Shantou 515041, China

**Keywords:** *Aquilaria sinensis* (Lour.) Gilg, artificial induction, chalcone synthase gene, chromone compound

## Abstract

Agarwood (gaharu) is a fragrant resin produced in the heartwood of resinous *Gyrinops* and *Aquilaria* species. Artificial agarwood samples were obtained from *Aquilaria sinensis* (Lour.) Gilg using formic acid (FA) stimulation combined with *Fusarium* sp. A2 inoculation. The relationship between the expression of chalcone synthase genes (*CHS*) and dynamic changes in chromone content was explored in resin-deposited parts of the trunks of *A. sinensis*. *CHS* gene expression levels were detected by qRT-PCR analysis. The chemical composition of agarwood obtained from the heartwood of *A. sinensis* before and within 1 year after induction was determined by GC-MS. After induction with FA stimulation combined with *F*. sp. A2 inoculation, the *CHS*1 gene showed relatively high expression, whereas the *CHS*2 gene showed low expression. The relative gene expression level of *CHS*1 peaked at 12 months, with a 153.1-fold increase, and the dominant period of the *CHS*2 gene expression was 10 months with a 14.13-fold increase. Moreover, chromones were not detected until after 2 months, and a large proportion of chromone compounds were detected after 4 months. Chromone content increased with time and peaked at 12 months. *CHS*1 gene expression was significantly correlated with 6-hydroxy-2-(2-phenylethyl)chromone accumulation, and *CHS*2 gene expression was significantly correlated with 5-hydroxy-6-methoxy-2-(2-phenylethyl)chromone accumulation. *CHS* gene expression was extremely sensitive to FA stimulation combined with *F*. sp. A2 inoculation and responded to late-onset injury. *CHS* genes expression also preceded the chromone accumulation. This work laid the foundation for studies on the mechanism by which genes regulate chromone biosynthesis pathways during the formation of agarwood resin in *A. sinensis*.

## 1. Introduction

Agarwood (gaharu) is a fragrant resin collected from resinous wood of *Gyrinops* and *Aquilaria* species. Agarwood is widely used as an effective traditional Chinese medicine and used to produce high-grade perfumes. Chinese agarwood is formed in the aromatic resinous wood of *Aquilaria sinensis* (Lour.) Gilg, mainly distributed in Guangdong Province, Hainan Province, and Guangxi Province. Owing to its high commercial value, agarwood is extensively harvested from forests. However, populations of the genus *Aquilaria* are considered threatened and have been listed in the International Union for Conservation of Nature (IUCN) Red List of Threatened Plants. In addition, all *Aquilaria* spp. are included in the List of Wild Plants under State Protection to protect the wild *Aquilaria* resources and ensure their sustainable use; thus, a permit is required before harvesting and trading these resources [1]. In forests, agarwood forms randomly at low frequency, approximately 7–10% of trees produce agarwood as a result of natural infections by fungi or wounding caused by wind, lightning, and gnawing by ants or insects [2]. These natural processes develop very slowly over decades. Most agarwood samples are currently induced through artificial methods, such as burning, injury, insect infestation, fungal pathogen infection, or inoculation either by chemicals or biological methods.

The mechanism of agarwood formation in trees has not yet been elucidated. A pathological response is speculated to occur as a result of fungal infection during agarwood formation [3,4,5,6,7]. Agarwood can be produced in *Aquilaria* as a response to endophytic fungal invasion [5,6,7,8]. Cui et al. [9,10] obtained three active strains i.e., *Microsphaeropsis* sp., *Xylaria* sp., and *Lasiodiplodia* sp., from 28 endophytic fungal strains in *A. sinensis*, and these strains significantly contribute to agarwood formation in *A. sinensis*. Tabata et al. [11], Ma et al. [12], Subehan et al. [13], Tian et al. [14], and Gao et al. [15] also isolated fungi, such as *Fusarium solani*, *Fusarium proliferatum*, and *Fusarium laseritum* from the resinous material of wounded *A. sinensis*. These studies showed that fungal infection is an effective method to induce agarwood production in *A. sinensis*. Feng [16] found that combined inoculation of two endophytic fungi (*Trichoderma reesei* and *Trichoderma koningii*) or inoculation of a single fungus (*Botryosphaeria rhodina*) into *A. sinensis* could induce production of high-quality agarwood. Guo et al. [17] found that inoculation of *F*. sp. A-11 and *Cephalosporium* sp. A-17 could induce *A. sinensis* to produce yellowish-brown substance, the constituents of which were similar to those of agarwood. Ma et al. [18] isolated *F. proliferatum* from the heartwood of *A. sinensis*, and agarwood trees were induced by fungal fermentation broth through pinhole instillation. As a result, agarwood formed within as short as 1 year, and agarwood production was 4 to 5 times higher than that obtained through other artificial induction methods. In addition, agarwood could be induced by chemical injury. Wang et al. [19] instilled formic acid, acetic acid, or their combination into the xylem of *A. sinensis*, and they found that the treatments could induce a defensive response in *A. sinensis*, resulting in a high production of agarwood.

Agarwood mainly contains sesquiterpenes and chromone derivatives [20]. In addition, a large proportion of fatty acid, fatty ester, and flavone derivatives are found in the trunks of *A. sinensis* [21,22,23]. Chalcone synthase is a key enzyme in the biosynthetic pathway of flavone compounds [24]. The structures of both flavone and 2-(2-phenylethyl)-chromone contain benzopyran; therefore, chromone formation in agarwood of *A. sinensis* may be related to flavone [25]. Metabolic regulation of gene expression while *Aquilaria* trees produce agarwood under stress conditions has been recently reported [26,27,28]. Wang et al. [29] successfully cloned the full-length *AsCHS*1 gene from wounded *A. sinensis* and found that the chalcone synthase gene (*CHS*) contains a conserved domain, which is a typical characteristic of *CHS*; additionally, they speculated that *AsCHS*1 proteins can catalyze chromone synthesis in *A. sinensis*. Cao et al. [30] cloned the promoter of *AsCHS*1 by using the genome walker strategy, and its function was preliminarily identified through a transient expression system to further investigate the expression and regulation mechanism of the product of *AsCHS*1 and elucidate the flavonoid accumulation in *A. sinensis* after wounding. Wang et al. [31] found that the chalcone synthase gene may contribute to the biosynthesis of 2-(2-phenylethyl)chromones. Ye et al. [32] performed transcriptome analysis of different parts of artificial agarwood from *A. sinensis* treated by formic acid and found that the expression level of the chalcone synthase gene in the agarwood part was much higher than that in the white wood part. This study used an agarwood-inducing method involving formic acid (FA) stimulation combined with *F.* sp. A2 inoculation to induce agarwood production in a 6-year-old *A. sinensis* [14,15,19]. The heartwoods of *A. sinensis* with or without resin were extracted before induction and within 1 year after induction, including seven time points. The relationship between *CHS* gene expression level and chromone content was tested and evaluated, and the results lay the foundation for studies on the mechanism regulating the chromone biosynthetic pathway during formation of agarwood resin in *A. sinensis*.

## 2. Results

### 2.1. Analysis of the Accumulation of Secondary Metabolite Compounds

**Figure 1:**
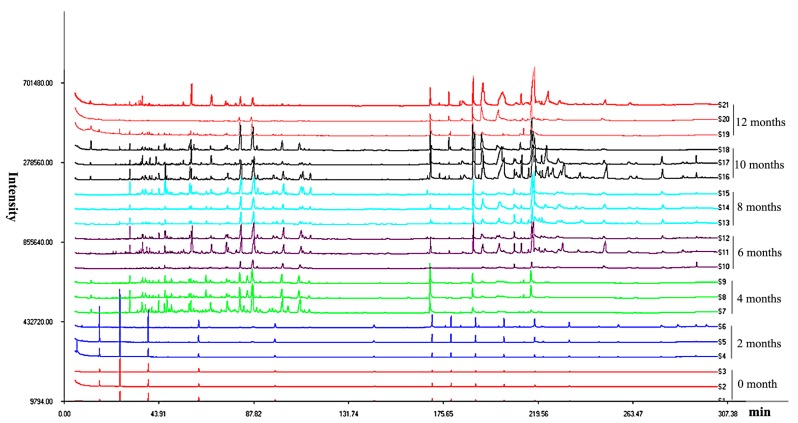
Overlapping GC-MS chromatogram for *A. sinensis* at pre- and post-induction by formic acid (FA) stimulation combined with *F*. sp. A2 inoculation at different time points. S1–S3: Before induction; S4–S6: 2 months after induction; S7–S9: 4 months after induction; S10–S12: 6 months after induction; S13–S15: 8 months after induction; S16–S18: 10 months after induction; S19–S21: 12 months after induction.

The three parallel samples collected at 0, 2, 4, 6, 8, 10, and 12 months after induction were analyzed by GC-MS, and the total ion chromatograms showed the changes in the composition and accumulation of compounds in artificial agarwood induced through FA stimulation combined with *F*. sp. A2 inoculation (Figure 1).

The components of 21 samples eluted within the total ion chromatogram were extracted in the Automatic Mass Spectral Deconvolution and Identification System (AMDIS), and sesquiterpenes and chromone compounds were identified by comparing the resolved mass spectra with those of the standards in the National Institute of Standards and Technology (NIST) Mass Spectral Library (05) (Table 1 and Table 2). The percentages of the total ion current were determined using the area normalization method. The main component was sesquiterpenes before 100 min and chromones after 169 min [33].

As shown in Table 1 and Table 2, 19 chromones and 16 sesquiterpenes were detected throughout the 12-month observation period. Chromones and sesquiterpenes were not identified in the artificial agarwood samples until 2 months after induction. A maximum of 23 secondary metabolite components with a relative percentage of 27.32% were identified in the samples after four months. The major chrome compounds (6.67%) included chromones 2-(2-phenylethyl) (2.20%), 6,7-dimethoxy-2-(2-phenylethyl) (1.95%), and 6-methoxy-2-(2-phenylethyl) (1.85%). A total of 29 secondary metabolite compounds were identified in the samples after six months and comprised 22.84% of chromones and 8.89% of sesquiterpenes. Up to 29 secondary metabolite components were identified in artificial agarwood samples induced by FA stimulation combined with *F*. sp. A2 after eight months, consisting of 42.29% chromones and 8.763% sesquiterpenes. A total of 13 chromones and 12 sesquiterpenes were identified in the artificial agarwood samples induced by FA stimulation combined with *F*. sp. A2 after 10 months. The accumulation of chromones reached the maximum in the agarwood samples artificially treated for 12 months, with a relative percentage of 44.87%.

Chromones were not detected in artificially induced trunks until after two months. After 4–12 months, a large proportion of chromone compounds, such as 2-(2-phenylethyl)chromone, 6-hydroxy-2-(2-phenylethyl)chromone, 6-methoxy-2-(2-phenylethyl)chromone, 5,8-dihydroxy-2-(2-phenylethyl)chromone, and 6-hydroxy-7-methoxy-2-(2-phenylethyl)chromone, were detected in these samples. Among these compounds, 6,7-dimethoxy-2-(2-phenylethyl)chromone showed high frequency with superior accumulation. Similar to chromones, sesquiterpenes were not detected in the trunks induced by artificial induction after two months. Most sesquiterpenes were detected at 4–12 months. Baimuxianal showed the highest frequency.

### 2.2. Relative Expression of Candidate Genes in Agarwood Samples Induced by FA Stimulation Combined with F. sp. A2 Inoculation

**Figure 2:**
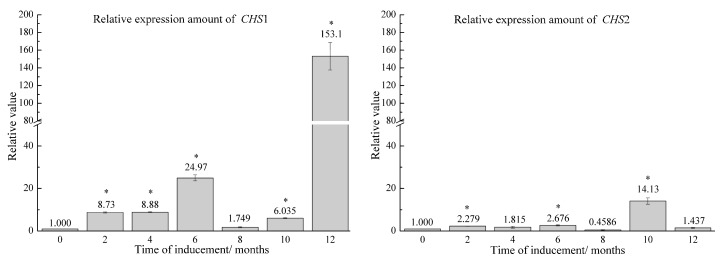
Relative expression amount of chalcone synthase genes (*CHS*) from artificial agarwood induced by FA stimulation combined with *F*. sp. A2 inoculation at different time points. * Means statistical significance of the difference between induced *A. sinensis* and healthy *A. sinensis* (*p* < 0.05).

To investigate the expression patterns of the genes related to chromone metabolism, we analyzed the expression level of *CHS*1 and *CHS*2 genes as shown in Figure 2. In the first 10 months, the relative expression level of *CHS*1 gene in the treated groups was below 25-fold. At the 12-month time point, the relative expression level increased markedly to 153.1-fold. The relative expression levels of *CHS*2 gene were low in the artificial agarwood induced by FA stimulation combined with *F*. sp. A2 inoculation from 0 to 8 months, ranging from 0.4585-fold to 2.676-fold. The largest increase in the expression levels of *CHS*2 gene was observed at 10 months with a 14.13-fold value. However, the relative expression level decreased to 1.437 after 12 months.

After being induced through FA stimulation combined with *F*. sp. A2 inoculation, the *CHS*1 gene showed a relatively high expression level, whereas that of the *CHS*2 gene was low. On the basis of *CHS* gene expression level, we speculated that the *CHS*1 gene was specifically expressed in phenylalanine pathways after induction through FA stimulation combined with *F*. sp. A2 inoculation.

### 2.3. Multivariate Analysis of Agarwood by Their Mass Ions and Identification

To explore the influence of the expression level of candidate genes in sesquiterpene or chromone biosynthesis, we determined the correlation between the relative expression level and the chemical compounds (sesquiterpenes and chromones) from different time points after induction (Table 3 and Table 4). Data were correlated using the statistical product and service solutions (SPSS) software (version 20.0, SPSS, Chicago, IL, USA). *CHS*1 gene expression level was significantly correlated to the accumulation of 6-hydroxy-2-(2-phenylethyl)chromone (A2), with a Pearson correlation coefficient approaching 1 (*p* < 0.01), and to 6-hydroxy-2-(2-phenylethyl)chromone (A3) and 6-hydroxy-7-methoxy-2-(2-phenylethyl)chromone (A6) accumulation (*p* < 0.05). *CHS*1 gene expression was also significantly correlated with eudesma-5,11(13)-dien-8,12-olide (A33) accumulation (*p* < 0.05). *CHS*2 gene expression level was only significantly correlated to the expression of 5-hydroxy-6-methoxy-2-(2-phenylethyl)chromone (A10) accumulation (*p* < 0.01).

**Figure 3:**
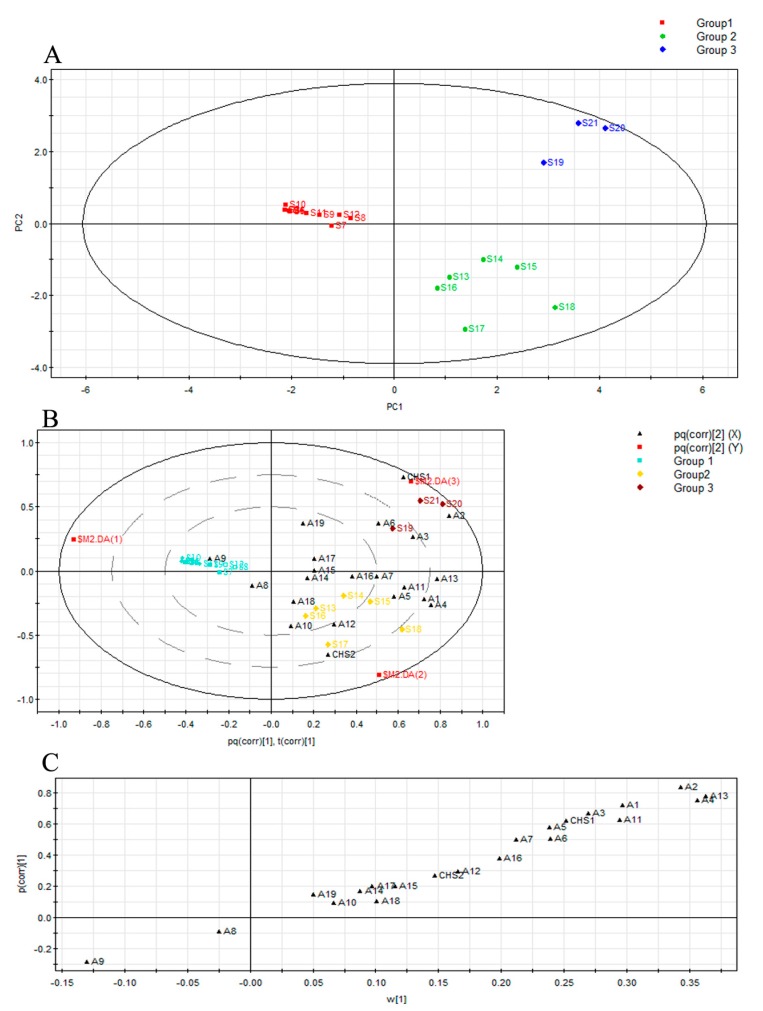
Orthogonal partial least squares discriminant analysis (OPLS-DA) results of mass ions of 21 batches of agarwood samples. (**A**), Loading Bi plot (**B**), and S-plot from OPLS-DA (**C**).

The orthogonal partial least squares discriminant analysis (OPLS-DA) (*R*^2^*X* = 0.652, *R*^2^*Y* = 0.922, *Q*^2^ (cum) = 0.622) score plot (Figure 3A) showed that artificial agarwood was evidently separated into three groups according to its induction period: samples obtained 0, 2, 4, and 6 months after induction, those obtained 8 and 10 months after induction, and those obtained 12 months after induction.

The peak area percentage of 19 chromone compounds and the relative *CHS* gene expression were examined by OPLS-DA using SIMCA-P 12.0 software, and a Loading Bi Plot was obtained (Figure 3B). The distribution of chromone compounds and *CHS* in the Loading Bi Plot reflects the corresponding contribution of the grouping. *CHS*1 gene expression was related to 6-hydroxy-2-(2-phenylethyl)chromone (A2), 6-hydroxy-2-(2-phenylethyl)chromone (A3), and 6-hydroxy-7-methoxy-2-(2-phenylethyl)chromone (A6). *CHS*2 gene expression was possibly related to 5-hydroxy-6-methoxy-2-(2-phenylethyl)chromone (A10) and 6-methoxy-2-[2-(3-methoxyphenyl) ethyl] chromone (A12). These results are essentially consistent with the result of the Pearson’s correlation analysis performed using SPSS software (Table 3).

S-plot was used to identify the compounds that significantly contributed in the grouping result (Figure 3C). The *p*(corr) is the correlation coefficient. 6,7-dimethoxy-2-(2-phenylethyl)chromone (A13, corr = 0.783161), 6-methoxy-2-(2-phenylethyl)chromone (A4, corr = 0.753625), and 6-hydroxy-2-(2-phenylethyl)chromone (A9, corr = −0.286252) substantially contributed to the grouping result. As shown above, the *p*(corr) values of chromones 6,7-dimethoxy-2-(2-phenylethyl) and 6-methoxy-2-(2-phenylethyl) were positive, whereas that of 6-hydroxy-2-(2-phenylethyl)chromone was false-positive.

## 3. Discussion

In this study, we used the FA stimulation combined with *F*. sp. A2 inoculation to produce artificial agarwood in the field. GC-MS combined with AMDIS and retention index (RI) correction index were used to study the variations in chemical components during agarwood formation. There is a clear trend in the approximate timing of chromone and sesquiterpene compounds increase, however, there seems to be a large difference in the GC-MS data. This may be due to individual variation caused by uncontrollable factors from field experiments. Chromone metabolites were not detected in the trunks in the first 2 months of induction; however, they were detected in the other four time points, and showed an increasing tendency. 6,7-dimethoxy-2-(2-phenylethyl)chromone showed the highest frequency. Lin et al. [34] detected 2-(2-phenylethyl)chromone compounds in agarwood after 1 year of artificial induction through fungal inoculation (*Melanotus flavolivens*) compared with that after a half-year induction. Qi et al. [24] found that chromone contents increased with time after artificial induction by *M. flavolivens*; the relative content of chromone in artificial agarwood also increased but not significantly.

The *CHS* gene is an inducible expression gene and can be induced after a plant is subjected to injury. *CHS* activation will be significantly enhanced and then *CHS* genes will be actively transcribed, transmitted, and amplified in vivo. *CHS* is regulated by a phenylalanine metabolism pathway to promote synthesis and release of chromone compounds [29]. Wang et al. [31] observed that chalcone synthase may regulate the biosynthesis of 2-(2-phenylethyl)chromones in response to salinity stress involved in *A. sinensis calli*. Ye et al. [32] performed the transcriptome analysis of different parts of artificial agarwood induced by formic acid. They found that the expression level of chalcone synthase in the agarwood part was much higher than that in the white wood part. After being induced by FA stimulation combined with *F*. sp. A2 inoculation, *CHS* gene expression level initially increased, then decreased, and finally increased. Our result showed that *CHS*1 gene was extremely sensitive to FA stimulation combined with *F*. sp. A2 inoculation, and *CHS*1 and *CHS*2 genes both responded to late-onset injury. Several studies showed that flavonoid accumulation paralleled the transcription level of change in the *CHS* gene [35,36,37]. Our study also showed the same trend in the agarwood samples induced by a chemical method plus fungal pathogen infection before 10 months. However, the *CHS*1 gene had the highest level of transcription at the last time point, while chromone accumulation reached a platform period. This suggests that the *CHS*1 gene may be turned into another biosynthesis pathway when the specific accumulation of chromone compounds reaches a steady state. Zhang et al. [38] demonstrated that the whole anthocyanin pathway was diverted to the synthesis of chlorogenic acid and its complexes when the expression of the *CHS* gene was severely repressed. Hoffman et al. [39] showed that the flavonoid pathway was shunted to the phenylpropanoid pathway when the *CHS* gene activity was reduced.

## 4. Materials and Methods

### 4.1. Source of Plant Materials

Materials were obtained from 6-year-old *A. sinensis* trees growing in a farm in Xinyi suburban district in Guangdong Province, China. These trees were identified as *A. sinensis* by Prof. Yan (College of Traditional Medicine, Guangdong Pharmaceutical University). *Fusarium* sp. A2 (EU781659) [40], provided and identified as *F*. sp. by Prof. Zhang (Guangdong Institute of Microbiology), was used to inoculate *A. sinensis* trees. Artificial agarwood from *A. sinensis* was induced through FA stimulation combined with *F.* sp. A2 inoculation through pinhole instillation according to the method of Gao et al. [33].

Induction was performed on the 24 June 2012, and wood chips containing embedded black resin were collected. The acquisition timeline was 0 month before induction and 2, 4, 6, 8, 10, and 12 months after induction. Three parallel samples were collected at each time point. Physical damage caused by manual removal of resin can induce agarwood production, so samples of different time points came from different *A. sinensis* trees individually. Samples from the same tree were divided into two portions. One portion was immediately wrapped in tinfoil and stored in liquid nitrogen for quantitative gene expression analysis, whereas the other portion was directly dried for GC-MS analysis.

### 4.2. Gas Chromatography Mass Spectrometry Analysis

All dried heartwood samples were cut into small pieces and filtered through 40-mesh sieves. The powder samples (0.5 g) were extracted in chloroform (10 mL, 24 h) at room temperature. The solvent was evaporated in a water bath (80 °C), then reconstituted to 2 mL chloroform and stored in a dark and air tight vial at 4 °C.

GC-MS analysis was performed using a GCMS QP-2010E (Shimadzu, Kyoto, Japan) equipped with a Rtx-5MS (Restek Corp., Bellefonte, PA, USA) capillary fused silica column (30 m × 0.25 mm I.D. × 0.25 μm film thickness), and operated in the electron ionization (EI) mode (70 eV). Helium was the carrier gas, with the flow rate of 1 mL/min. The operating parameters were the temperature program of 90 °C for 4 min, ramp of 2.5 °C/min up to 160 °C (5 min), then increased to 180 °C with a 0.3 °C/min heating vamp, kept for 5 min, and then ramp of 2.0 °C/min up to 200 °C. Subsequently it was increased to 230 °C with a 1 °C/min heating vamp, and kept at 230 °C for 120 min. A 1 μL sample solution was injected. The injections were performed in a 1:30 split ratio at 230 °C. The *m*/*z* values were recorded in the range of 50–500 amu. A 1 μL C_10_–C_31_ sample was injected separately and was run in the same program as the heartwood samples.

### 4.3. Quantitative Real-Time PCR (qRT-PCR)

To analyze gene expression levels, total ribonucleic acid (RNA) was isolated from heart wood samples as previously described [41]. Reverse transcription was performed using a ReverTra Ace qCR RT kit Master Mix with gDNA Remover (TOYOBO, Osaka, Japan) with specific primers (Supplementary Table S1). The specificity of the oligonucleotide sequences, in relation to its annealing efficiencies, was evaluated using the Primer 5.0 program in advance. A fragment of the glyceraldehyde-3-phosphate dehydrogenase (*GADPH*) gene was also amplified as a blank control [42]. The qPCR analysis was performed using an ABI 7500 Real-Time PCR System (Applied Biosystems, Foster City, CA, USA), using a THUNDERBIRD SYBR qPCR Mix (Toyobo, Osaka, Japan). The Ct (threshold cycle) was used to measure the starting copy numbers of each target gene, and was detected by the ABI 7500 Real-Time PCR System. Relative quantitation of each target gene expression level was performed using the comparative 2^−ΔΔCt^ method [43]. All experiments were conducted in triplicate. 

### 4.4. Data Processing and Statistics Analysis

The components were identified based on the comparison of their retention indices and mass spectra as previously described [33,42]; moreover, we used NIST MS search 2.0 with the database of NIST 05 after elution within the total ion chromatogram (TIC) extracted in the AMDIS. Retention indices were calculated using a series of n-alkanes (C_10_–C_31_). Correlations between chemical components and expression of the *CHS* gene were analyzed by the Pearson’s correlation analysis of SPSS (20.0 for Windows). Peak-area percentage of chromone compounds and relative expression of *CHS* were examined by OPLS-DA using SIMCA-P 12.0 software.

## 5. Conclusions

2-(2-Phenylethyl)chromone compounds, which are the main characteristic components of agarwood, are closely related to the plant self-defense response. Defensive substances, such as sesquiterpenes and chromones with bacteriostatic activity, will be produced when a plant is subjected to injury or fungal infection. These defensive substances can protect *A. sinensis* from further injury. Recent investigations have revealed that in the early stage of injury, a plant mainly releases a large proportion of volatile compounds derived from lipoxygenase and then releases chromone compounds later. Chromone content increases with continuous injury [44]. In this study, we used an agarwood inducing method involving FA stimulation combined with *F.* sp. A2 inoculation to induce agarwood production in individual *A. sinensis* trees. The relationship between the expression of chalcone synthase gene (*CHS*) and dynamic changes in chromone content was explored by using qRT-PCR and GC-MS analysis. The present study showed that *CHS* gene expression preceded the chromone accumulation after induction through FA stimulation combined with *F*. sp. A2 inoculation. Chromone compounds were not detected in the artificially induced trunk until after 2 months, even when *CHS* gene expression had already begun. Chromone compounds were detected 4 months after induction. The release of chromone compounds was closely related to *CHS* expression in agarwood formation. Therefore, we can regulate *CHS* expression to produce chromone compounds in the future, which would lay the foundation for highly efficient artificial agarwood production and elucidation of the mechanism of agarwood formation in *A. sinensis.*

## Figures and Tables

**Table 1:** Dynamic change of chromone compounds from *A. sinensis* pre- and post-treatment by FA stimulation combined with *F*. sp. A2 inoculation ^a^.

No.	RT ^b^	RI ^c^	Chemical Name	Formula	Relative Percentage Content/% (Sample Number of Each Compound Can Be Retrieved)						
					0 Month	2 Months	4 Months	6 Months	8 Months	10 Months	12 Months
A1	169.693	2297	2-(2-phenylethyl)chromone	C_17_H_14_O_2_	-	-	2.2000	1.0533	2.6300	4.1600	3.6667
A2	178.191	2423.6	6-hydroxy-2-(2-phenylethyl)chromone	C_17_H_14_O_3_	-	-	0.0433	0.3300	0.3533	0.4733	1.3867
A3	184.57	2513.5	6-hydroxy-2-(2-phenylethyl)chromone	C_17_H_14_O_3_	-	-	-	0.3167	0.2700	0.6267	1.3100
A4	189.177	2578.4	6-methoxy-2-(2-phenylethyl)chromone	C_17_H_14_O_3_	-	-	1.8467	1.6767	6.9333	6.4700	5.8067
A5	193.68	2641.8	6-methoxy-2-(2-phenylethyl)chromone	C_17_H_14_O_3_	-	-	0.3267	2.2867	2.6067	3.8633	3.1433
A6	193.908	2641.6	6-hydroxy-7-methoxy-2-(2-phenylethyl)chromone	C_18_H_16_O_3_	-	-	0.1200	-	1.9733	0.2900	4.5767
A7	200.424	2736.9	6-hydroxy-2-(2-phenylethyl)chromone	C_17_H_14_O_3_	-	-	-	1.3733	5.0600	3.2133	4.8733
A8	201.506	2752.1	6-hydroxy-2-[2-(4'-methoxyphenyl)ethyl]chromone	C_18_H_16_O_4_	-	-	-	2.0500	1.3167	-	-
A9	203.394	2778.7	6-hydroxy-2-(2-phenylethyl)chromone	C_17_H_14_O_3_	-	-	-	0.6533	-	-	-
A10	203.424	2779.1	5-hydroxy-6-methoxy-2-(2-phenylethyl)chromone	C_17_H_14_O_3_	-	-	-	0.2433	-	0.6500	-
A11	211.355	2890.8	6-methoxy-2-[2-(3-methoxyphenyl)ethyl]chromone	C_19_H_18_O_4_	-	-	0.2500	0.6200	1.2767	0.8100	1.1600
A12	214.793	2939.2	6-methoxy-2-[2-(3-methoxyphenyl)ethyl]chromone	C_19_H_18_O_4_	-	-	-	-	0.6500	0.1833	-
A13	216.212	2959.2	6,7-dimethoxy-2-(2-phenylethyl)chromone	C_19_H_18_O_4_	-	-	1.9500	6.0767	12.6967	11.6833	13.2467
A14	218.373	2989.7	5,8-dihydroxy-2-[2-(4′-met hoxyphenethyl)]chromone	C_18_H_16_O_5_	-	-	-	2.9933	1.7667	1.0500	1.4100
A15	220.064	3013.5	6,8-dihydroxy-2-[2-(3′-methoxy-4′-hydroxyl phenylethyl)]chromone	C_18_H_16_O_5_	-	-	-	0.4733	0.9467	-	0.4833
A16	222.57	3048.8	6-hydroxy-7-methoxy-2-(2-phenylethyl)chromone	C_18_H_16_O_3_	-	-	-	1.5267	1.8400	2.3667	2.7067
A17	228.351	3130.2	6-hydroxy-2-[2-(4′-methoxyphenyl)ethyl]chromone	C_18_H_16_O_4_	-	-	-	0.7833	0.9000	-	0.6900
A18	233.746	3206.2	6,8-dihydroxy-2-[2-(3′-methoxy-4'-hydroxyl phenylethyl)]chromone	C_18_H_16_O_5_	-	-	0.03	-	1.0700	-	-
A19	262.132	3606.1	6,8-dihydroxy-2-[2-(3′-methoxy-4′-hydroxyl phenylethyl)]chromone	C_18_H_16_O_5_	-	-	-	0.3867	-	-	0.4100
Relative percentage content of total chromonetration (*n* = 3)					-	-	6.767 ± 2.30	22.84 ± 6.60	42.29 ± 3.52	35.84 ± 10.27	44.87 ± 19.44

^a^ Identification was made according to comparison of resolved mass spectra with those of standards in the Mass Library Database. ^b^ Retention time. ^c^ Retention index. - Not detected in the sample.

**Table 2:** Dynamic change of sesquiterpene compounds from *A. sinensis* pre- and post-treatment by FA stimulation combined with *F*. sp. A2 inoculation ^a^.

No.	RT ^b^	RI ^c^	Chemical Name	Formula	Relative Percentage Content/ % (Sample Number of Each Compound Can Be Retrieved)						
					0 Month	2 Months	4 Months	6 Months	8 Months	10 Months	12 Months
A20	30.411	1575.4	Isoaromadendrene epoxide	C_15_H_24_O	-	-	0.8900	0.5267	0.2433	0.2733	0.2233
A21	33.98	1620.6	Aromadendrene oxide-(1)	C_15_H_24_O	-	-	0.5067	0.1200	-	0.0300	0.0467
A22	34.736	1628.4	Agarospirol	C_15_H_26_O	-	-	1.0733	0.0633	-	0.0333	0.3200
A23	36.234	1644	Guaiol	C_15_H_26_O	-	-	2.9500	0.6400	0.0933	0.2100	0.2567
A24	41.098	1694.6	Santalol	C_15_H_24_O	-	-	0.1533	0.0767	-	0.1500	0.0667
A25	42.373	1705.4	Aromadendrene oxide-(2)	C_15_H_24_O	-	-	0.1167	0.0967	0.0767	-	-
A26	43.736	1715.3	2-(4a,8-Dimethyl-1,2,3,4,4a,5,6,7-octahydro-naphthalen-2-yl)-prop-2-en-1-ol	C_15_H_24_O	-	-	0.2500	0.4567	0.0433	0.2200	0.1233
A27	46.384	1734.4	Longipinocarvone	C_15_H_22_O	-	-	0.3733	0.4733	0.0733	0.0200	-
A28	46.719	1737	Germacrone	C_15_H_22_O	-	-	0.2767	0.4067	0.2267	0.1533	0.1000
A29	47.54	1742.8	Viridiflorol	C_15_H_26_O	-	-	0.9233	0.4233	0.0800	0.1933	-
A30	49.743	1758.7	γ-Gurjunenepoxide-(2)	C_15_H_24_O	-	-	0.7100	0.2233	0.0467	-	-
A31	58.158	1817.5	Baimuxinal	C_15_H_24_O_2_	-	-	4.5400	2.5200	0.3733	1.7333	1.5033
A32	67.806	1865.4	Longifolenaldehyde	C_15_H_24_O	-	-	0.8233	0.3467	0.1133	1.1700	0.2367
A33	81.632	1927.4	Eudesma-5,11(13)-dien-8,12-olide	C_15_H_20_O_2_	-	-	1.6833	-	1.0867	1.5867	4.0500
A34	81.939	1928.6	Velleral	C_15_H_20_O_2_	-	-	-	-	5.9500	-	3.8500
A35	89.478	1957	Vellerdiol	C_15_H_24_O_2_	-	-	3.4367	-	0.3567	0.1700	0.3100
A36	100.903	2000.1	6-(1-Hydroxymethylvinyl)-4,8a-dimethyl-3,5,6,7,8,8a-hexahydro-1H-naphthalen-2-one	C_15_H_22_O_2_	-	-	2.8433	2.5167	-	-	-
Relative percentage content of total sesquiterpenetration (*n* = 3)					-	-	21.55 ± 3.63	8.890 ± 2.46	8.763 ± 1.56	5.943 ± 1.73	11.09 ± 9.45

^a^ Identification was made according to comparison of resolved mass spectra with those of standards in the Mass Library Database. ^b^ Retention time. ^c^ Retention index. - Not detected in the sample.

**Table 3:** Correlative analysis between expression of *CHS* genes and accumulation of chromone compounds ^a^.

		A1	A2	A3	A4	A5	A6	A7	A8	A9	A10	A11	A12	A13	A14	A15	A16	A17	A18	A19
*CHS*1	Pearson correlation	0.372	0.922 **	0.873 *	0.261	0.338	0.870 *	0.467	−0.229	−0.074	−0.261	0.426	−0.338	0.436	0.165	0.201	0.536	0.358	−0.275	0.749
	Bilateral significance	0.467	0.009	0.023	0.617	0.512	0.024	0.35	0.663	0.889	0.618	0.4	0.512	0.387	0.755	0.703	0.273	0.485	0.598	0.087
*CHS*2	Pearson correlation	0.498	−0.002	0.168	0.323	0.518	−0.312	0.055	−0.291	−0.108	0.953 **	0.013	−0.042	0.246	−0.06	−0.488	0.342	−0.492	−0.327	−0.266
	Bilateral significance	0.315	0.997	0.751	0.533	0.292	0.548	0.918	0.576	0.839	0.003	0.98	0.938	0.639	0.91	0.326	0.507	0.322	0.527	0.61

^a^ Statistic significance was determined by the Pearson correlation analysis with the SPSS software (20.0). A1–A19 from Table 1. ** Means significantly correlated at 0.01 level (double side). * Means significantly correlated at 0.05 level (double side).

**Table 4:** Correlative analysis between expression of *CHS* genes and accumulation of sesquiterpene compounds ^a^.

		A20	A21	A22	A23	A24	A25	A26	A27	A28	A29	A30	A31	A32	A33	A34	A35	A36
*CHS*1	Pearson correlation	−0.174	−0.156	0.079	−0.174	−0.038	−0.39	−0.065	−0.266	−0.243	−0.331	−0.257	−0.036	−0.238	0.821 *	0.347	−0.165	−0.229
	Bilateral significance	0.742	0.767	0.882	0.742	0.943	0.445	0.903	0.611	0.642	0.521	0.624	0.946	0.65	0.045	0.5	0.755	0.662
*CHS*2	Pearson correlation	−0.111	−0.188	−0.242	−0.184	0.576	−0.428	0.195	−0.243	−0.114	−0.063	−0.252	0.03	0.786	−0.004	−0.433	−0.203	−0.238
	Bilateral significance	0.835	0.722	0.644	0.727	0.231	0.397	0.711	0.643	0.83	0.905	0.63	0.955	0.064	0.994	0.391	0.699	0.649

^a^ Statistic significance was determined by the Pearson correlation analysis with the SPSS software (20.0). A20–A42 from Table 2.* Means significantly correlated at 0.05 level (double side).

## Supplementary Materials

The supplementary materials are available online.
