# Supplementary material for: Relationship between Expression of Chalcone Synthase Genes and Chromones in Artificial Agarwood induced by Formic Acid Stimulation Combined with Fusarium sp. A2 Inoculation
Source: Molecules. 2017 Apr 25;22(5):686. doi: 10.3390/molecules22050686 (PMC6154532; doi:10.3390/molecules22050686)
Supplement: Supplementary file 1 [file molecules-22-00686-s001.pdf]

Supplementary Table S1. Chemical compounds from *A. sinensis* pre- and post-treated by FA stimulation combined with *F.* sp. A2 inoculation [a].

No.	No. in this study	RT[b]	RI[c]	Chemical name	Formula	Relative percentage content/ %（sample number of each compound can be retrieved）						
						0 month	2 months	4 months	6 months	8 months	10 months	12months
1	-	12.385	1249.3	2-Butanone, 4-phenyl-	$C_{10}H_{12}O$	-	-	0.46	0.0533	0.04	0.7333	0.84
2	-	25.262	1500	Tridecane,2-methyl-	$C_{14}H_{3}O$	5.0267	1.2933	-	-	-	-	-
3	-	27.848	1539.1	Dodecane,2,6,11-trimethyl	$C_{15}H_{32}$	5.1267	0.44	0.0367	0.0433	0.0333	-	
4	A20	30.411	1575.4	Isoaromadendrene epoxide	$C_{15}H_{24}O$	-	-	0.89	0.5267	0.2433	0.2733	0.2233
5	-	31.791	1610	Diethyl phthalate	$C_{12}H_{14}O_{4}$	-	0.08	0.0167	-	-	-	-
6	A21	33.98	1620.6	Aromadendrene oxide-(1)	$C_{15}H_{24}O$	-	-	0.5067	0.12	-	0.03	0.0467
7	A22	34.736	1628.4	Agarospirol	$C_{15}H_{26}O$	-	-	1.0733	0.0633	-	0.0333	0.32
8	A23	36.234	1644	Guaiol	$C_{15}H_{26}O$	-	-	2.95	0.64	0.0933	0.21	0.2567
9	A24	41.098	1694.6	Santalol	$C_{15}H_{24}O$	-	-	0.1533	0.0767	-	0.15	0.0667
10	A25	42.373	1705.4	Aromadendrene oxide-(2)	$C_{15}H_{24}O$	-	-	0.1167	0.0967	0.0767	-	-
11	-	42.562	1600	Hexadecane	$C_{16}H_{34}$	5.71	0.29	-	-	-	-	-
12	A26	43.736	1715.3	2-(4a,8-Dimethyl-1,2,3,4,4a,5,6,7-octahydro-naphthalen-2-yl)-prop-2-en-1-ol	$C_{15}H_{24}O$	-	-	0.25	0.4567	0.0433	0.22	0.1233
13	A27	46.384	1734.4	Longipinocarvone	$C_{15}H_{22}O$	-	-	0.3733	0.4733	0.0733	0.02	-
14	A28	46.719	1737	Germacrone	$C_{15}H_{22}O$	-	-	0.2767	0.4067	0.2267	0.1533	0.1
15	A29	47.54	1742.8	Viridiflorol	$C_{15}H_{26}O$	-	-	0.9233	0.4233	0.08	0.1933	-
16	A30	49.743	1758.7	γ-Gurjunenepoxide-(2)	$C_{15}H_{24}O$	-	-	0.71	0.2233	0.0467	-	-
17	-	57.848	1814.2	5,5,8a-Trimethyldecalin-1-one	$C_{13}H_{22}O$	-	-	1.1767	-	-	0.9267	-
18	A31	58.158	1817.5	Baimuxinal	$C_{15}H_{24}O_{2}$	-	-	4.54	2.52	0.3733	1.7333	1.5033
19	A32	67.806	1865.4	Longifolenaldehyde	$C_{15}H_{24}O$	-	-	0.8233	0.3467	0.1133	1.17	0.2367
20	A33	81.632	1927.4	Eudesma-5,11(13)-dien-8,12-olide	$C_{15}H_{20}O_{2}$	-	-	1.6833	-	1.0867	1.5867	4.05
21	A34	81.939	1928.6	Velleral	$C_{15}H_{20}O_{2}$	-	-	-	-	5.95	-	3.85
22	-	87.326	1933	Dibutyl phthalate	$C_{16}H_{22}O_{4}$	-	6.4933	-	-	-	-	-
23	A35	89.478	1957	Vellerdiol	$C_{15}H_{24}O_{2}$	-	-	3.4367	-	0.3567	0.17	0.31
24	-	92.442	1968.2	n-Hexadecanoic acid	$C_{16}H_{32}O_{2}$	-	-	-	-	-	0.0533	0.0667
25	-	96.491	1984.1	Acetic acid, 3-hydroxy-6-isopropenyl-4,8a-dimethyl-1,2,3,5,6,7,8,8a-octahydronaphthalen-2-yl ester	$C_{17}H_{26}O_{3}$	-	-	1.01	1.1067	1.03	0.4733	0.0733

26	A36	100.903	2000.1	6-(1-Hydroxymethylvinyl)-4,8a-dimethyl-3,5,6,7,8,8a-hexahydro-1H-naphthalen-2-one	$C_{15}H_{22}O_2$	-	-	2.8433	2.5167	-	-	-
27	A1	169.693	2297	2-(2-phenylethyl)chromone	$C_{17}H_{14}O_2$	-	-	2.2	1.0533	2.63	4.16	3.6667
28	-	173.815	2355.6	9-Octadecenamide, (Z)-	$C_{18}H_{35}NO$	-	-	-	-	-	0.1767	0.5733
29	-	176.544	2700	Heptacosane	$C_{27}H_{56}$	6.66	0.9233	-	-	-	-	-
30	A2	178.191	2423.6	6-hydroxy-2-(2-phenylethyl)chromone	$C_{17}H_{14}O_3$	-	-	0.0433	0.33	0.3533	0.4733	1.3867
31	-	183.607	2400	Tetracosane	$C_{24}H_{50}$	8.62	1.1333	-	-	-	-	-
32	A3	184.57	2513.5	6-hydroxy-2-(2-phenylethyl)chromone	$C_{17}H_{14}O_3$	-	-	-	0.3167	0.27	0.6267	1.31
33	-	187.189	2548	1,2-Benzenedicarboxylic acid, mono(2-ethylhexyl) ester	$C_{16}H_{22}O_4$	-	1.58	0.0233	-	0.1367	0.12	-
34	A4	189.177	2578.4	6-methoxy-2-(2-phenylethyl)chromone	$C_{17}H_{14}O_3$	-	-	1.8467	1.6767	6.9333	6.47	5.8067
35	-	192.155	2700	Heptacosane	$C_{27}H_{56}$	8.4733	1.7133	0.6733	-	-	-	-
36	A5	193.68	2641.6	6-methoxy-2-(2-phenylethyl) chromone	$C_{17}H_{14}O_3$	-	-	0.3267	2.2867	2.6067	3.8633	3.1433
37	A6	193.908	2641.8	6-hydroxy-7-methoxy-2-(2-phenylethyl) chromone	$C_{18}H_{16}O_3$	-	-	0.12	-	1.9733	0.29	4.5767
38	A7	200.424	2736.9	6-hydroxy-2-(2-phenylethyl)chromone	$C_{17}H_{14}O_3$	-	-	-	1.3733	5.06	3.2133	4.8733
39	-	201.13	2700	Heptacosane	$C_{27}H_{56}$	5.76	0.9433	1.51	-	-	-	-
40	A8	201.506	2752.1	6-hydroxy-2-[2-(4'-methoxyphenyl)ethyl]chromone	$C_{18}H_{16}O_4$	-	-	-	2.05	1.3167	-	-
41	A9	203.394	2778.7	6-hydroxy-2-(2-phenylethyl)chromone	$C_{17}H_{14}O_3$	-	-	-	0.6533	-	-	-
42	A10	203.424	2779.1	5-hydroxy-6-methoxy-2-(2-phenylethyl) chromone	$C_{17}H_{14}O_3$	-	-	-	0.2433	-	0.65	-
43	-	208.573	2851	13-Docosenamide, (Z)-	$C_{22}H_{43}NO$	-	-	-	2.63	1.0567	0.6833	-
44	-	210.941	2700	Heptacosane	$C_{27}H_{56}$	-	1.21	-	-	-	-	-
45	-	211.132	2800	Octacosane	$C_{28}H_{58}$	4.8267	-	-	-	-	-	-
46	A11	211.355	2890.8	6-methoxy-2-[2-（3’-methoxyphenyl）ethyl]chromone	$C_{19}H_{18}O_4$	-	-	0.25	0.62	1.2767	0.81	1.16

47	-	213.182	2752	Squalene	$C_{30}H_{50}$	0.03	-	0.3333	0.1567	0.21	-	-
48	A12	214.793	2939.2	6-methoxy-2-[2-(3-methoxyph enyl)ethyl]chromone	$C_{19}H_{18}O_4$	-	-	-	-	0.65	0.1833	-
49	A13	216.212	2959.2	6,7-dimethoxy-2-(2-phenyleth yl)chromone	$C_{19}H_{18}O_4$	-	-	1.95	6.0767	12.6967	11.6833	13.2467
50	A14	218.373	2989.7	5,8-dihydroxy-2-[2-(4’-metho xyphenethyl)]chromone	$C_{18}H_{16}O_5$	-	-	-	2.9933	1.7667	1.05	1.41
51	A15	220.064	3013.5	6,8-dihydroxy-2-[2-(3'-metho xy-4'-hydroxyl phenylethyl)] chromone	$C_{18}H_{16}O_5$	-	-	-	0.4733	0.9467	-	0.4833
52	-	221.18	2700	Heptacosane	$C_{27}H_{56}$	3.73	3.14	-	-	-	-	-
53	A16	222.57	3048.8	6-hydroxy-7-methoxy-2-(2-ph enylethyl)chromone	$C_{18}H_{16}O_3$	-	-	-	1.5267	1.84	2.3667	2.7067
54	A17	228.351	3130.2	6-hydroxy-2-[2-(4'-methoxyp henyl)ethyl]chromone	$C_{18}H_{16}O_4$	-	-	-	0.7833	0.9	-	0.69
55	A18	233.746	3206.2	6,8-dihydroxy-2-[2-(3'-metho xy-4'-hydroxyl phenylethyl)] chromone	$C_{18}H_{16}O_5$	-	-	0.03	-	1.07	-	-
56	-	247.57	3100	Hentriacontane	$C_{31}H_{64}$	5.7533	11.12	-	-	-	-	-
57	A19	262.132	3606.1	6,8-dihydroxy-2-[2-(3'-metho xy-4'-hydroxyl phenylethyl)] chromone	$C_{18}H_{16}O_5$	-	-	-	0.3867	-	-	0.41
58	-	292.587	4035.1	Stigmast-4-en-3-one	$C_{29}H_{48}O$	24.9833	23.0867	-	1.26	0.9033	0.4967	0.07

[a] Identification was made according to comparison of resolved mass spectra with those of standards in Mass Library Database.
[b] retention time
[c] retention index

Supplementary Table S2. Specific primers of genes used in qRT-PCR assays.

Primer name	Forward primer（5'-3'）	Reverse primer（5'-3'）	Length/bp
GAPDH	CTGGTATGGCATTCCGTGTA	AACCACATCCTCTTCGGTGTA	161
*CHS*1	TCACCAGGAGCGATCACAT	GGCGACCAGTAGTCAGCAAT	139
*CHS*2	CCAACAGCGAGCACATGACC	TTCTTTGCCCAACTTCGGGATC	194
